# Impact of Endovascular Treatment on the Development of Post-Thrombotic Syndrome in Iliac and Iliofemoral Deep Vein Thrombosis

**DOI:** 10.3390/jcm14176280

**Published:** 2025-09-05

**Authors:** Gabriel Puche Palao, Javier Trujillo-Santos, Sonia Otálora, Leticia Guirado-Torrecillas, Javier Pagán-Escribano, Pablo Demelo-Rodríguez

**Affiliations:** 1 Department of Internal Medicine, Hospital Universitario Virgen de Arrixaca, Vereda de la Barca No. 12, 30120 Murcia, Spain; sdpotal@hotmail.com (S.O.); leticia.guiradotorrecillas@gmail.com (L.G.-T.); 2 Department of Internal Medicine, Hospital General Universitario Santa Lucía, 30202 Murcia, Spain; javier.trujillosantos@gmail.com; 3 Department of Internal Medicine, Hospital General Universitario Morales Meseguer, 30008 Murcia, Spain; pagan02468@gmail.com; 4 Venous Thromboembolism Unit, Hospital General Universitario Gregorio Marañón, 28007 Madrid, Spain; pbdemelo@hotmail.com; 5 School of Medicine, Universidad Complutense de Madrid, 28040 Madrid, Spain; 6 Instituto de Investigación Sanitaria Gregorio Marañón, 28007 Madrid, Spain

**Keywords:** deep venous thrombosis, thrombolysis, thrombectomy, endovascular, anticoagulation, post-thrombotic syndrome, residual thrombosis

## Abstract

**Background:** Post-thrombotic syndrome (PTS) is a frequent complication of deep vein thrombosis (DVT), with significant clinical and quality-of-life implications. Endovascular techniques have emerged as potential strategies to reduce PTS severity in selected patients, though evidence remains inconclusive. **Methods:** We conducted a multicenter, retrospective study including 176 patients with iliac or iliofemoral DVT from four hospitals in Murcia, Spain. Patients were treated either with anticoagulation alone (*n* = 121) or with endovascular techniques followed by anticoagulation (*n* = 55). The primary outcome was the presence of PTS at 12 months, defined by the presence of ≥5 signs/symptoms from the Villalta scale. Multivariate analysis was performed to identify independent predictors of PTS. **Results:** No significant differences were observed in the overall prevalence of PTS between the endovascular and anticoagulation-only groups at 12 months (25.4% vs. 23.2%, *p* = 0.63). However, edema and skin hyperpigmentation were significantly more frequent in the anticoagulation-only group. Patients treated with endovascular techniques had lower rates of residual thrombosis at follow-up (34.6% vs. 53.9%, *p* = 0.03). Multivariate analysis identified residual thrombosis (OR 6.3, 95% CI 2.74–14.5), persistently elevated D-dimer (OR 3.26, 95% CI 1.1–9.7), and age (OR 1.02, 95% CI 1.003–1.042) as independent predictors of PTS. Mortality was significantly higher in the anticoagulation-only group, largely driven by a higher cancer prevalence. **Conclusions:** Endovascular techniques did not significantly reduce the overall incidence of PTS but were associated with lower rates of specific signs such as edema and hyperpigmentation. Residual thrombosis remains a key predictor of PTS and may represent a modifiable therapeutic target.

## 1. Introduction

The majority of venous thromboembolism (VTE) patients are treated with anticoagulation to prevent recurrences, thrombus progression, and pulmonary embolism. However, despite adequate treatment, 20–50% of patients with deep vein thrombosis (DVT) develop post-thrombotic syndrome (PTS) within two years, depending on the classification criteria used [1]. Severe PTS, including venous ulcers, affects 5–10% of patients [2], underscoring the need for strategies to mitigate its substantial medical, social, and economic burden.

Residual venous obstruction and valvular reflux following DVT contribute to sustained venous hypertension, leading to inflammation, tissue hypoxia, edema, fibrosis, and, in severe cases, ulceration [3]. Under normal conditions, venous pressure ranges from 80 to 90 mmHg when standing and decreases to approximately 22 mmHg during ambulation. Venous valve damage or obstruction disrupts this mechanism, resulting in pathological venous hypertension. Although thrombus recanalization occurs in roughly 50% of patients within the first three months, residual thrombus may persist in up to half of cases after three years [4]. Early thrombus resolution could help minimize valvular damage and subsequent reflux, although accurate quantification of residual obstruction and reflux remains challenging [5].

Endovascular techniques have emerged as promising strategies to reduce the severity of PTS, especially in high-risk patients. Interventions such as catheter-directed thrombolysis (CDT), pharmaco-mechanical thrombolysis (PMT), and ultrasound-accelerated CDT (USCDT) aim to restore venous patency and reduce PTS risk following proximal DVT, without significantly increasing hemorrhagic complications [6,7]. These approaches may offer advantages over anticoagulant alone by preventing chronic symptoms that impair quality of life. Early intervention is essential, as thrombin generation during the acute phase can damage venous valves [8]. The European Society of Vascular Surgery recommends CDT or PMT for iliofemoral DVT who exhibit rapid thrombus progression despite anticoagulation, worsening symptoms (Villalta score > 10), low bleeding risk, and symptom onset within 14 days [9]. In line with this, the 2025 ESVM Guidelines on Interventional Treatment of Venous Thromboembolism recommend that catheter-based therapy (CBT) should be considered in patients with severe symptoms due to iliofemoral and/or iliocaval acute DVT, with a class II recommendation and level of evidence A [10].

The aim of this study is to evaluate whether CDT or thrombectomy followed by anticoagulation more effectively reduces the incidence of PTS in patients with highly symptomatic proximal DVT involving iliac or iliofemoral veins, compared to anticoagulation alone.

## 2. Methods

### 2.1. Study Design and Patient Population

This was a retrospective, multicenter cohort study with an analytical component, based on structured review of hospital database. The study was conducted in the Region of Murcia, Spain, and included population data from four hospitals, covering a total of 1,013,508 inhabitants.

The study population consisted of consecutive male and female patients aged 18 years or older with a diagnosis of acute deep vein thrombosis (DVT) affecting the iliac region. Patient selection was based on a review of hospital databases between January 2016 and June 2022. Medical records were reviewed independently by two investigators to confirm eligibility and extract clinical data.

Inclusion criteria were (1) Age ≥ 18 years; (2) confirmed diagnosis of DVT involving the iliac vein, verified by lower extremity venous Doppler ultrasound or computed tomography venography; (3) availability of data for at least 75% of the primary study variables; and (4) a minimum follow-up of 12 months from diagnosis. The exclusion criteria were as follows: (1) DVT without iliac involvement; (2) participation in clinical trials involving investigational drugs; (3) lack or withdrawal of informed consent; and (4) contraindications to anticoagulant therapy.

All patients were treated in specialized thrombosis outpatient clinics at the participating centers. Treatment and follow-up were performed by the attending physicians according to local protocols and clinical guidelines. All procedures were conducted in accordance with the ethical standards of the Declaration of Helsinki and were approved by the Clinical Research Ethics Committee of HGURS (CEI Code 1042021). Informed consent, either written or verbal, was obtained from all patients or their legal representatives, according to the protocols of each center. All eligible cases during the study period were included; no formal sample size calculation was performed.

### 2.2. Procedures

Once iliac DVT was confirmed, relevant variables were recorded in a structured registry. Baseline sociodemographic data, acute-phase treatments, and long-term management strategies were documented. Treatment decisions were made at the discretion of the treating physicians following local protocols and clinical guidelines and were not standardized across centers. Endovascular techniques were considered for patients with severe symptoms lasting less than 14 days. The final decision to perform thrombectomy was made by a multidisciplinary team composed of the responsible clinical team and the interventional vascular radiology department. Endovascular procedures were performed using two different catheter systems, AngioJet^®^ Thrombectomy System (Boston Scientific Corporation, Marlborough, MA, USA) or Aspirex^®^ S Mechanical Thrombectomy System (Becton, Dickinson and Company [BD], Franklin Lakes, NJ, USA). Fibrinolytic therapy consisted of either alteplase, administered at a dose of 0.5 mg/kg infused over 4 h, or urokinase, at a dose of 4400 U/kg/hour. No immediate complications were observed during the procedures. All interventions were carried out by experienced interventional vascular radiologists.

All patients received low-molecular-weight heparin during the acute phase, followed by either DOACs, VKAs, or continuation of LMWH. Subtherapeutic INR was defined as a time in therapeutic range (TTR) < 50% during the first 3 months of VKA therapy. In addition, some patients were treated with sulodexide, and compression stockings were used in selected cases. The sulodexide regimen consisted of 250 lipasemic units (LSU) administered orally twice daily for a minimum of 2 to 3 months, as recommended by Gloviczki P et al. [11]. Below-knee elastic compression stockings with a pressure of 20–40 mmHg were prescribed from the time of diagnosis and recommended for at least the first 6 months. Adherence was defined as use on ≥5 days per week and was assessed during follow-up visits.

In our cohort, antiplatelet therapy was prescribed exclusively in patients who underwent endovascular procedures due to venous stent placement. No cases of antiplatelet use were related to secondary prevention of coronary artery disease or ischemic stroke, as none of the patients had prior cardiovascular indications for aspirin therapy. At the time the study was conducted, no firm recommendations existed regarding the use of antiplatelet agents after endovascular venous interventions, and to date, there remains ongoing controversy about whether antiplatelet therapy should be systematically employed in this setting.

Follow-up was conducted in outpatient settings by the responsible physicians. Data were collected through clinical interviews and/or medical records from the participating centers.

### 2.3. Follow-Up and Outcomes

The primary outcome was the presence of PTS at 12 months. Clinical variables recorded at follow-up visits included those from the Villalta scale: pain, cramps, heaviness, pruritus, paresthesia, edema, skin induration, hyperpigmentation, collateral venous circulation, erythema, calf tenderness, and venous ulceration (either active or healed). Due to incomplete recording of symptom severity in many medical records, a diagnosis of PTS was assumed when five or more signs or symptoms listed in the Villalta scale were present.

### 2.4. Statistical Analysis

Baseline characteristics were described using appropriate statistical methods. The Kolmogorov–Smirnov test was used to assess the normality of continuous variables. Normally distributed variables were expressed as mean ± standard deviation (SD), while non-normally distributed variables were reported as median and interquartile range (IQR, P25–P75). Categorical variables were expressed as frequency (*n*) and percentage (%). Comparisons between groups were made using the Chi-square test (χ^2^) or Fisher’s exact test for categorical variables. Continuous variables were compared using Student’s *t*-test or ANOVA for normally distributed data, and the Mann–Whitney U test or Kruskal–Wallis test for non-normally distributed data. A two-sided *p*-value < 0.05 was considered statistically significant.

Additionally, a multivariate logistic regression analysis was performed to identify independent predictors of post-thrombotic syndrome at 12 months. For the multivariate model, variables with *p* < 0.10 in the univariate analysis were selected. Odds ratios (OR) with 95% confidence intervals (95% CI) were calculated. Missing data were handled by complete case analysis.

Statistical analyses were performed using IBM SPSS Statistics for MacOS, Version 26.0 (IBM Corp., New York, NY, USA).

## 3. Results

### 3.1. Patient Demographics and Baseline Characteristics

A total of 176 patients with iliac DVT were included in the study. Of these, 55 were treated with endovascular techniques followed by anticoagulation, and 121 received anticoagulation alone. The majority of patients were female (57.3%), with a median age of 56.2 years (IQR 41.2–72.9). Patients in the anticoagulation-only group were significantly older than those in the endovascular group (62.0 vs. 41.3, *p* < 0.001). Cardiovascular risk factors were significantly more prevalent in the anticoagulation-only group, which also had higher rates of chronic obstructive pulmonary disease (COPD), heart failure, cerebrovascular disease, and dementia. In contrast, 52.7% of patients in the endovascular group were diagnosed with May–Thurner syndrome. Baseline characteristics are summarized in Table 1.

All patients received low-molecular-weight heparins (LMWH) during the acute phase. Regarding long-term anticoagulation, 45.5% were treated with vitamin K antagonists (VKAs), 30.1% with LMWH, and 24.4% with direct oral anticoagulants (DOACs). Patients in the endovascular group were more likely to be treated with DOACs (49.1% vs. 13.2%, *p* < 0.001) and with antiplatelet agents after discontinuing anticoagulation (73.1% vs. 26.9%, *p* < 0.001).

Detailed treatment data are shown in Table 2.

### 3.2. Endovascular Procedures

Among patients treated with endovascular techniques, 41.8% (23/55) underwent pharmaco-mechanical thrombolysis, 21.8% (12/55) received catheter-directed thrombolysis alone, and 36.3% (20/55) underwent catheter-based thrombectomy alone. Popliteal vein access was used in 98.2% of cases. A prophylactic inferior vena cava filter was placed in 18.1% of patients before the procedure. In total, 61.8% (34/55) of endovascular patients received venous stenting. Minor bleeding complications occurred in four patients (7.2%), with no major bleeding events reported.

The median time from symptom onset to intervention was 7 days (IQR 4.0–11.5). In patients undergoing thrombectomy, AngioJet^®^ rheolysis system was used in 46.67% of cases, and Aspirex^®^ aspiration thrombectomy in 53.3%. In all patients undergoing catheter-directed thrombolysis, urokinase was the thrombolytic agent of choice.

### 3.3. Outcomes

No significant differences were observed in the overall incidence of post-thrombotic syndrome (PTS) between the anticoagulation-only group and the endovascular group at 12 months (23.2% vs. 25.5%, *p* = 0.63). However, the prevalence of two PTS-related symptoms was significantly higher in the anticoagulation-only group: lower-limb edema (54.5% vs. 40.0%, *p* = 0.019) and skin hyperpigmentation (37.2% vs. 18.2%, *p* = 0.004). No significant differences were found for other symptoms included in the Villalta scale. Detailed information regarding symptoms and signs of PTS in both groups are shown in Table 3. The proportion of missing data for each Villalta scale item is presented in Supplementary Table S3.

To address the potential influence of anticoagulant type, we performed a sensitivity analysis excluding patients who received long-term DOAC therapy. In this restricted cohort, the differences between groups in terms of edema and skin induration persisted, whereas no significant differences were observed for other post-thrombotic syndrome variables (Supplementary Table S1). Moreover, we performed a sensitivity analysis excluding patients on VKA with sub-therapeutic INR, and we found significant differences between groups regarding paresthesia, pretibial edema, and skin induration (Supplementary Table S2).

At 12-month follow-up, residual thrombosis was present in 53.9% of patients in the anticoagulation-only group, compared to 34.6% in the endovascular group. This difference was statistically significant (*p* = 0.03).

In the multivariate logistic regression model adjusted for age, sex, prior DVT, body weight, residual thrombosis, D-dimer at diagnosis, and persistently elevated D-dimer levels, independent predictors of PTS at 12 months were residual thrombosis (OR 6.3; 95% CI 2.74–14.5), persistently elevated D-dimer (OR 3.26; 95% CI 1.1–9.7), and age (OR 1.02; 95% CI 1.003–1.042). All variables included in the multivariate model had a *p*-value < 0.10 in exploratory comparisons between patients with and without PTS.

A significant difference in mortality was observed between the groups: 26.4% (32/121) in the anticoagulation-only group vs. 0% (0/55) in the endovascular group (*p* < 0.001). This difference may be partially explained by a higher prevalence of malignancy at diagnosis in the anticoagulation group (30.6% vs. 7.3%, *p* = 0.001), including a greater proportion of patients with metastatic disease (18.2% vs. 0%, *p* = 0.001).

Recurrent venous thromboembolism occurred in 10.7% (13/121) of patients in the anticoagulation-only group and in 7.3% (4/55) of the endovascular group (*p* = 0.470). There were no significant differences in recurrence type (*p* = 0.424). Most recurrences were ipsilateral in both groups (83.3% in the anticoagulation group and 100% in the endovascular group; *p* = 0.290).

## 4. Discussion

Our study did not find significant differences between the two groups in terms of overall diagnosis of PTS at the 12-month follow-up. However, analysis of individual signs and symptoms revealed key differences. Patients treated with anticoagulation alone had a significantly higher prevalence of lower-limb edema and skin hyperpigmentation, suggesting that while endovascular techniques may not prevent PTS globally, they may attenuate specific features associated with its clinical severity.

These findings align with those from the ATTRACT trial, in which patients receiving pharmacomechanical thrombolysis had a lower incidence of moderate-to-severe PTS (Villalta score ≥ 10) compared to those on anticoagulation alone (18% vs. 24%), although the study did not detail which specific symptoms accounted for this difference [12]. Notably, only 57% of the 692 participants in ATTRACT had iliac involvement, a factor associated with more severe PTS. A subsequent sub-analysis of patients with iliofemoral DVT confirmed that endovascular strategies reduced PTS severity and improved quality of life compared to anticoagulation alone [13].

Despite improvements in select symptoms, the overall prevalence of PTS remained similar between groups, even though the anticoagulation-only cohort was significantly older and had a higher burden of comorbidities, including malignancy. This suggests that the benefit of endovascular techniques may be partially counteracted by other mechanisms contributing to PTS. Moreover, residual thrombosis at 12 months, a known predictor of PTS, was significantly less common in the endovascular group (34.6% vs. 53.9%, *p* = 0.03), but this reduction did not translate into lower overall PTS rates.

In our cohort, catheter-directed thrombolysis (CDT) combined with thrombectomy using AngioJet^®^ was the most commonly employed strategy, and it was associated with a lower rate of edema at 12 months. These results are consistent with those from Vedantham et al., who demonstrated that CDT, including rheolytic techniques, improved symptom severity and reduced edema in the short term. However, other symptoms such as heaviness, pain, and collateral circulation showed no significant differences between groups, despite a notably lower rate of residual thrombosis in the endovascular group [14]. This observation raises questions about the complex pathophysiology of PTS. It is possible that endothelial injury from the intervention, venous inflammation, or procedural factors such as required immobility after catheter insertion could offset some of the benefits of reduced thrombus burden. A possible mechanistic explanation for the symptomatic improvement observed in specific domains (e.g., edema and hyperpigmentation) despite the lack of an overall reduction in PTS rates may relate to partial restoration of venous outflow after endovascular therapy. Even in the presence of residual thrombosis, re-establishing iliac patency can reduce venous hypertension and microvascular congestion, thereby alleviating edema and skin changes. In contrast, symptoms more closely associated with chronic microvascular damage or inflammatory responses, such as heaviness or paresthesia, may not be equally responsive to improved venous drainage. This divergence supports the concept that PTS is a heterogeneous syndrome with multifactorial pathophysiology, in which macrovascular recanalization and microvascular/inflammatory damage contribute to different domains of the clinical spectrum. The observed reduction in edema and hyperpigmentation should not be interpreted as evidence of overall therapeutic efficacy, as these improvements do not necessarily translate into comprehensive disease control. A plausible explanation is that irreversible valvular damage and persistent microvascular dysfunction may continue to drive the pathophysiological processes underlying chronic venous disease. These mechanisms could limit the long-term impact of the intervention despite symptomatic relief in selected clinical manifestations.

Previous studies suggested long-term benefits of endovascular techniques in reducing PTS rates at 12, 24, and 36 months, and even up to 5 years of follow-up [15,16,17]. Haig et al. reported sustained improvements after CDT, and similar findings were observed in early analyses of the CaVenT trial [18]. However, subsequent large randomized clinical trials, such as ATTRACT and CAVA, did not demonstrate a long-term reduction in PTS incidence following endovascular techniques, leading to increased skepticism regarding the utility of these interventions in routine practice [19,20].

In our study, 52.7% of patients in the endovascular group were diagnosed with May–Thurner syndrome, and 61.8% received venous stenting during the index procedure. Both conditions are closely linked to the pathophysiology and prognosis of PTS. However, due to the observational design and sample size limitations, their individual impact on the development of PTS could not be assessed independently. May–Thurner syndrome is a recognized anatomical risk factor for venous stasis and recurrent thrombosis [21]. While stent placement may improve long-term venous patency and symptom relief, it may also carry procedural risks or complications that influence outcomes. In a recent Delphi consensus document, there was agreement that “stenting can be considered in patients with acute symptomatic DVT of compressive cause with involvement of at least the iliac vein, who present severe symptoms and good previous functional status, and in those with persistent severe symptoms despite initial anticoagulation treatment, after thrombolysis (mechanical or pharmaco-mechanical)” [22]. These criteria largely overlap with the profile of patients included in our study who underwent endovascular therapy, supporting the rationale behind current clinical decision-making in selected cases of iliofemoral DVT.

Our study adds to the evidence by offering real-world data derived from specialized thrombosis clinics and reflecting usual clinical decision-making processes. It provides a detailed analysis of individual symptoms and signs of PTS and uses a standardized treatment approach, with consistent use of CDT and thrombectomy devices (primarily AngioJet^®^ and Aspirex^®^) and urokinase as the thrombolytic agent. Furthermore, our findings support the role of early thrombus removal in reducing residual thrombosis and selected symptoms, although its impact on PTS diagnosis per se remains unclear.

A validated risk stratification tool for early-phase DVT could enhance the selection of candidates for endovascular therapies. Although Villalta scoring was originally developed to assess PTS after the acute phase, its early use may help identify patients who would benefit most from intervention. Prior studies have shown that patients with Villalta scores >10 at diagnosis derive greater benefits from CDT [1,23,24,25]. Therefore, future clinical trials should consider using acute-phase Villalta scoring as an inclusion criterion for endovascular strategies. Additionally, differences in PTS incidence among patients receiving various direct oral anticoagulants (DOACs), as suggested by post hoc analyses of ATTRACT, merit further investigation [26]. In our study, we conducted sensitivity analyses excluding patients on long-term DOAC therapy and those with subtherapeutic INR; in both cases, the results remained largely unchanged (Supplementary Tables S1 and S2), suggesting that the type of anticoagulant had no significant impact.

Future research should also systematically incorporate quality of life as a core outcome measure, as highlighted by the CaVenT trial, which showed that patients who developed post-thrombotic syndrome experienced significantly poorer quality of life compared to those who did not develop PTS [27]. This underscores the importance of not only preventing PTS but also minimizing its clinical manifestations that impair daily functioning and well-being. Moreover, comprehensive cost-effectiveness analyses of endovascular techniques should be prioritized, given the growing economic burden of chronic venous disease. Previous studies have reported conflicting results regarding the cost-efficiency of catheter-directed thrombolysis (CDT) [28]. For instance, while the CaVenT trial reported a modest gain in quality-adjusted life years (QALYs) favoring CDT, the ATTRACT trial found that CDT was not cost-effective for patients with proximal DVT, largely due to the procedure’s cost and the lack of significant reduction in overall PTS rates. These results underscore the critical need for refined patient selection criteria to identify individuals who would benefit the most from these resource-intensive interventions [29]. In this context, several ongoing clinical trials (ChiCTR-INR-16009090; IRCT201108035625N3; NCT02959801) are currently exploring the effectiveness and safety of various endovascular modalities, their impact on long-term functional outcomes, and their economic viability in broader populations. Expanding and refining research in this field are essential not only to improve clinical efficacy and patient safety, but also to optimize cost-effectiveness, particularly in healthcare systems facing increasing demands. Future studies should aim to define the patient subgroups in whom these interventions can meaningfully reduce the burden of PTS, enhance long-term outcomes, and contribute to sustained improvements in the comprehensive management of venous thromboembolic disease.

In our cohort, overall mortality was higher in the anticoagulation group. This difference is largely explained by baseline characteristics: a higher prevalence of cancer and metastases, a higher median age, and a greater burden of comorbidities. As mortality was not a primary endpoint of this study, these results should be interpreted with caution to avoid overstating a potential survival benefit of endovascular intervention.

This study has several limitations. First, the retrospective design of our study may inherently introduce selection bias, as patient inclusion was not randomized and could have been influenced by clinical decision-making processes. Second, baseline differences between the comparison groups may act as potential confounders, limiting the ability to establish causal inferences. Although we adjusted for relevant covariates, the possibility of residual confounding cannot be completely excluded. Additionally, anticoagulant regimens differed significantly between groups, with more frequent use of DOACs in the endovascular group, which may have impacted PTS outcomes. Third, although the Villalta scale was used to define PTS, we lacked detailed data on the severity of individual symptoms, preventing calculation of full Villalta score. Instead, a simplified definition based on the presence of five or more signs/symptoms was applied consistently across the cohort. This approach may underestimate the true prevalence of PTS but reflects the limitations often encountered in routine clinical documentation. In addition, in Supplementary Table S3, we present the exact proportions of missing data for each individual item of the Villalta scale. Fourth, patients were included only if they had completed 12 months of follow-up, which may introduce selection bias by excluding those with early complications or incomplete care pathways. Fifth, we acknowledge that quality of life was not assessed using validated instruments such as the VEINES-QOL/Sym scale. Future studies should incorporate standardized measures of quality of life to provide a more comprehensive evaluation. Finally, it should be acknowledged that endovascular therapy is a resource-intensive intervention. While our study was not designed to formally evaluate cost-effectiveness or quality of life, these aspects are critical to guide the real-world implementation of such strategies. Future prospective trials incorporating economic analyses and patient-reported outcomes will be essential to establish the overall value of these interventions.

## 5. Conclusions

In this real-world multicenter study, endovascular techniques for the treatment of iliac or iliofemoral DVT did not significantly reduce the overall prevalence of post-thrombotic syndrome at 12 months compared to anticoagulation alone. However, these interventions were associated with a lower prevalence of residual thrombosis and with improvements in specific symptoms of venous insufficiency, particularly edema and hyperpigmentation. The findings highlight the need for better risk stratification and patient selection to identify those who may derive long-term benefit from endovascular therapy.

## Figures and Tables

**Table 1:** Baseline clinical and sociodemographic characteristics of the groups in our cohort.

	Total (*n*: 176)	Anticoagulation Only (*n*: 121)	Endovascular Techniques (*n*: 55)	*p*-Value
Sex—*n* (%)				
Female	101 (57.4)	65 (53.7)	36 (65.5)	0.1954
Age (years)—Median (IQR)	56.22 (41.2–72.9)	62.02 (50.5–78.2)	41.29 (33.3–50.7)	<0.001
Weight (kg)—Median (IQR)	73 (63.2–83)	72 (50.53–78.26)	76 (65–85.2)	0.054
Ethnicity				0.041
- Caucasian	162 (92)	114 (94.2)	48 (87.3)	
- Arab	5 (2.8)	1 (0.8)	4 (7.3)	
- Black	4 (2.3)	1 (0.8)	3 (5.5)	
- Latin American	2 (1.1)	2 (1.7)	0	
- Other	3 (1.7)	3 (2.5)	0	
Cardiovascular Risk Factors—*n* (%)				
Hypertension	63 (35.8)	59 (48.8)	4 (7.3)	<0.001
Dyslipidemia	52 (29.5)	49 (40.5)	3 (5.5)	<0.001
Diabetes	24 (13.6)	24 (19.8)	1	<0.001
Smoking	61 (34.7)	46 (38)	15 (27.3)	0.001
Comorbidities—*n* (%)				
COPD	13 (7.4)	13 (10.7)	0	0.01
Heart failure	11 (6.3)	11 (9.1)	0	0.02
Atrial fibrillation	4 (2.3)	4 (3.3)	0	0.171
Ischemic heart disease	11 (6.3)	10 (8.3)	1 (1.8)	0.099
Peripheral vascular disease	13 (7.4)	12 (9.9)	1 (1.8)	0.055
Cerebrovascular disease	13 (7.4)	13 (10.7)	0	0.011
Dementia	9 (5.1)	9 (7.4)	0	0.037
Chronic kidney disease	7 (4)	7 (5.8)	0	0.068
Connective tissue disease	7 (4)	3 (2.5)	4 (7.3)	0.135
HIV infection	1 (0.6)	1 (0.8)	0	0.784
Liver disease	1 (0.6)	1 (0.8)	0	0.499
Recent major bleeding	2 (1.1)	2 (1.7)	0	0.336
Concomitant Conditions at Diagnosis—*n* (%)				
Cava Agenesis	5 (2.8)	3 (2.5)	2 (3.6)	0.675
May–Thurner syndrome	37 (21)	8 (6.6)	29 (52.7)	<0.001
Concurrent pulmonary embolism—*n* (%)	40 (33.1)	21 (38.2)	21 (65.6)	0.657
VTE risk factors—*n* (%)				
Cancer	41 (23.29)	37 (30.6)	4 (7.3)	0.001
Metastatic cancer	22 (12.5)	22 (18.2)	0	0.001
Surgery last 2 months	27 (15.3)	25 (20.7)	2 (3.6)	0.004
Immobilized for 4 days or more for a non-surgical reason	64 (36.3)	55 (45.5)	9 (16.4)	<0.001
Prior VTE	24 (13.6)	14 (11.7)	10 (18.2)	0.245
Recent travel	7 (3.9)	3 (2.5)	4 (7.3)	0.131
Hormonal treatment in the last 2 months	28 (15.9)	13 (10.7)	15 (27.3)	0.005
Pregnancy	2 (1.13)	2 (1.7)	0	0.338
Varicose veins in lower extremities	20 (11.36)	11 (9.1)	9 (16.4)	0.159
Puerperium	1 (0.5)	0	1 (1.8)	0.137

IQR, Interquartile Range; COPD, Chronic Obstructive Pulmonary Disease; HIV, Human Immunodeficiency Virus; VTE: venous thromboembolism.

**Table 2:** Long-term anticoagulant therapy options in patients undergoing endovascular techniques vs. anticoagulation alone.

	Total (*n*: 176)	Endovascular Techniques (*n*: 55)	Anticoagulation Only (*n*: 121)	*p*-Value
Vitamin K antagonist	80 (45.5)	22 (40)	58 (47.9)	0.327
Low-molecular-weight heparin	53 (30.1)	6 (11.1)	47 (38.8)	<0.001
Direct Oral anticoagulants	43 (24.4)	27 (49.2)	16 (13.2)	<0.001
Antiplatelet therapy	19 (10.8)	19 (34.5)	0	<0.001
Antiplatelet therapy after stopping anticoagulation	26 (39.4)	19 (73.1)	7 (17.5)	<0.001
Sulodexide	23 (13.1)	13 (24.1)	10 (8.3)	0.005
Compression stockings	86 (48.9)	42 (79.2)	44 (36.7)	<0.001

**Table 3:** Prevalence of signs and symptoms related to post-thrombotic syndrome at 12 months of follow-up. Values are presented as *n*/N (%), where N indicates the number of patients with available data for each variable at 12 months.

	Endovascular Techniques (*n* = 55)	Anticoagulation Only (*n* = 121)	*p*-Value
***n*/N (%)**	**12 month**		
Pain	27/48 (56.3)	55/106 (51.9)	0.61
Cramps	10/49 (20.4)	22/93 (23.7)	0.660
Heaviness	31/48 (64.6)	56/95 (58.9)	0.514
Pruritus	13/47 (27.7)	23/88 (26.1)	0.849
Paresthesia	8/46 (17.4)	27/91 (29.7)	0.120
Pretibial edema	22/48 (45.8)	66/100 (66)	0.019
Skin induration	12/48 (25)	41/101 (40.6)	0.063
Hyperpigmentation	10/48 (20.8)	45/100 (45)	0.004
Venous ectasia	15/47 (31.9)	43/101 (42.6)	0.216
Pain on calf compression	15/49 (30.6)	29/100 (29)	0.839
Venous ulcer	0/46	5/102 (4.9)	0.127

## Data Availability

Data are not publicly available due to ethical and institutional restrictions related to patient confidentiality. Anonymized data may be made available from the corresponding author upon reasonable request.

## Supplementary Materials

The following supporting information can be downloaded at: https://www.mdpi.com/article/10.3390/jcm14176280/s1, Table S1: Sensitivity analysis excluding patients on long-term DOAC therapy: post-thrombotic syndrome outcomes at 12 months; Table S2: Sensitivity analysis excluding patients with subtherapeutic INR: post-thrombotic syndrome outcomes at 12 months; Table S3: Proportion of missing data for each Villalta scale item at 12-month follow-up.
