# Supplementary material for: Impact of Endovascular Treatment on the Development of Post-Thrombotic Syndrome in Iliac and Iliofemoral Deep Vein Thrombosis
Source: J Clin Med. 2025 Sep 5;14(17):6280. doi: 10.3390/jcm14176280 (PMC12429572; doi:10.3390/jcm14176280)
Supplement: Supplementary file 1 [file jcm-14-06280-s001.zip › jcm-3792595-supplementary.pdf]

Journal of
Clinical Medicine

MDPI

Supplementary Table S1. Sensitivity analysis excluding patients on long-term DOAC therapy: post-thrombotic syndrome outcomes at 12 months.

Variable	Endovascular Techniques	Anticoagulation Only	p value
**n/N (%)**			
**Pain**	11/24 (45.8%)	47/91 (51.6%)	0.391
**Cramps**	4/25 (16.0%)	21/79 (26.6%)	0.212
**Heaviness**	14/24 (58.3%)	50/80 (62.5%)	0.445
**Pruritus**	7/24 (29.2%)	22/74 (29.7%)	0.558
**Paresthesia**	5/23 (21.7%)	23/77 (29.9%)	0.316
**Pretibial edema**	**11/24 (45.8%)**	**59/85 (69.4%)**	**0.031**
**Skin induration**	**6/25 (24.0%)**	**38/85 (44.7%)**	**0.050**
**Hyperpigmentation**	10/25 (40.0%)	43/86 (50.0%)	0.257
**Venous ectasia**	12/23 (52.2%)	40/86 (46.5%)	0.402
**Pain on calf compression**	7/25 (28.0%)	28/86 (32.6%)	0.432
**Venous ulcer**	0/22 (0.0%)	4/88 (4.5%)	0.404

Supplementary Table S2. Sensitivity analysis excluding patients with subtherapeutic INR: post-thrombotic syndrome outcomes at 12 months.

Variable	Endovascular Techniques (n=51)	Anticoagulation Only (n=105)	p value
**Pain**	24/45 (53.3%)	26/91 (28.6%)	0.451
**Cramps**	8/45 (17.8%)	18/79 (22.8%)	0.338
**Heaviness**	28/45 (62.2%)	48/81 (59.3%)	0.448
**Pruritus**	11/43 (25.6%)	16/75 (21.3%)	0.378
**Paresthesia**	**5/43 (11.6%)**	**22/78 (28.2%)**	**0.028**
**Pretibial edema**	**19/45 (42.2%)**	**55/85 (64.7%)**	**0.011**
**Skin induration**	10/44 (22.7%)	29/86 (33.7%)	0.137
**Hyperpigmentation**	**8/44 (18.2%)**	**35/86 (40.7%)**	**0.007**
**Venous ectasia**	12/44 (27.3%)	32/86 (37.2%)	0.175
**Pain on calf compression**	11/45 (24.4%)	22/85 (25.9%)	0.517
**Venous ulcer**	0/43 (0.0%)	5/88 (5.7%)	0.132

Supplementary Table S3. Proportion of missing data for each Villalta scale item at 12-month follow-up.

Villalta scale item	Available Endovascular (n=55)	Missing Endovascular, n (%)	Available Anticoagulation (n=121)	Missing Anticoagulation, n (%)
**Pain**	48	7 (12.7%)	106	15 (12.4%)
**Cramps**	49	6 (10.9%)	93	28 (23.1%)
**Heaviness**	48	7 (12.7%)	95	26 (21.5%)
**Pruritus**	47	8 (14.5%)	88	33 (27.3%)
**Paresthesia**	46	9 (16.4%)	91	30 (24.8%)
**Pretibial edema**	48	7 (12.7%)	100	21 (17.4%)
**Skin induration**	48	7 (12.7%)	101	20 (16.5%)
**Hyperpigmentation**	48	7 (12.7%)	100	21 (17.4%)
**Venous ectasia**	47	8 (14.5%)	101	20 (16.5%)
**Pain on calf compression**	49	6 (10.9%)	100	21 (17.4%)
**Venous ulcer**	46	9 (16.4%)	102	19 (15.7%)

n, number of patients. Percentages are calculated with respect to the total number of patients in each treatment group.
